# Chemosensation and Evolution of *Drosophila* Host Plant Selection

**DOI:** 10.1016/j.isci.2019.100799

**Published:** 2019-12-23

**Authors:** Robert R.H. Anholt

**Affiliations:** 1 Department of Genetics and Biochemistry and Center for Human Genetics, Clemson University, Greenwood, SC 29646, USA

**Keywords:** Biological Sciences, Genetics, Evolutionary Biology

## Abstract

The ability to respond to chemosensory cues is critical for survival of most organisms. Among insects, *Drosophila melanogaster* has the best characterized olfactory system, and the availability of genome sequences of 30 *Drosophila* species provides an ideal scenario for studies on evolution of chemosensation. Gene duplications of chemoreceptor genes allow for functional diversification of the rapidly evolving chemoreceptor repertoire. Although some species of the genus *Drosophila* are generalists for host plant selection, rapid evolution of olfactory receptors, gustatory receptors, odorant-binding proteins, and cytochrome P450s has enabled diverse host specializations of different members of the genus. Here, I review diversification of the chemoreceptor repertoire among members of the genus *Drosophila* along with co-evolution of detoxification mechanisms that may have enabled occupation of diverse host plant ecological niches.

## Graphical Abstract

**Figure:**
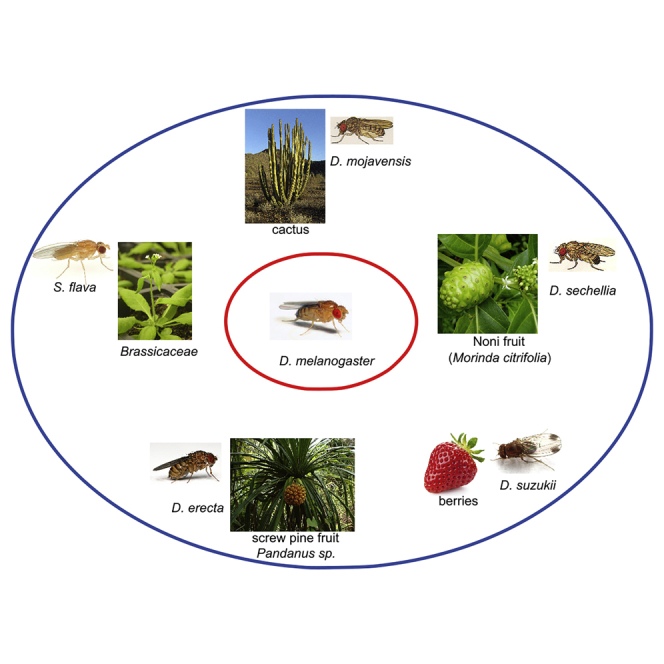

## Behaviors as a Platform for Evolution

Behaviors, i.e., interactions of an organism with its environment, are the ultimate expression of the nervous system. Behaviors mediate interactions between the environment and conspecific and heterospecific individuals, which are essential for survival and reproductive success. Thus, behaviors provide a substrate for natural selection and “survival of the fittest.” From a genetics perspective, behaviors are quantitative traits because their manifestation is determined by multiple segregating genes and influenced by the environment. Genetic variation is mediated through *mutation-selection balance*, as well as genetic *drift* within a population and *gene flow* between populations. Selective forces, often driven by environmental pressures, act upon genetic variation within a population, leading to fixation or shifts in frequencies of alleles (Slatkin, 1987, Orr and Betancourt, 2001, Yeaman and Otto, 2011, Huang et al., 2016, Alexander et al., 2017). Behavioral traits are prime targets for natural selection, because these traits are often highly variable and plastic, and behaviors associated with survival and reproduction are determinants of *fitness*. *Drosophila* provides an exceptional model system for understanding the evolution of behavior (Glossary).

Glossary Accessory gland: Male glands that provide products to sustain the sperm and include seminal fluid proteins that affect female physiology and behavior after mating

Arista: A feather-like appendage that emanates from the base of the antenna

Circadian: Biological rhythms that occur in approximately 24-hour periods

***cis***-regulatory elements: Regions of non-coding DNA that regulate the transcription of neighboring genes

Copy number variants: Insertions, deletions, and duplications of segments of DNA that vary among individuals within a population

Directional selection: A type of natural selection in which a single phenotype is favored, causing the allele frequency to continuously shift in one direction thereby favoring extreme values over intermediate values

Drift: Random variation in allele frequency caused by sampling in finite populations

Evolvability: The capacity to undergo adaptive evolution

Fitness: The genetic contribution of an individual to the next generation

Functional redundancy: The situation in which multiple genes contribute in equivalent ways and can substitute for one another in generating the phenotype

Gene duplication: Duplication of a region of DNA that contains a gene, resulting in an extra copy of that gene

Gene flow: The introduction of genetic material by interbreeding from one population of a species to another, thereby changing the composition of the gene pool of the receiving population

Gene ontology enrichment analyses: A bioinformatics technique for interpreting whether sets of genes with similar functional classifications are overrepresented in the dataset.

Ionotropic receptors: Membrane-bound receptor proteins that respond to ligand binding by opening an ion channel

Knockout: An animal from whose genome a gene has been removed

Loss-of-function allele: An allele in which a mutation has occurred so that the altered gene product lacks the molecular function of the wild-type gene

Molecular response profiles: The spectrum of molecules that can elicit a biological response from a receptor

Mutation-selection balance: The equilibrium between the rate at which mutations arise and their elimination by natural selection

Neofunctionalization: Acquisition of a novel function

Odorant: A molecule that carries an odor

Oviposition: Egg laying

Paralogs: Genes that derive from the same ancestral gene

Phylogeny: The relationship among different groups of organisms based on their evolutionary history

Plasticity: The ability of one genotype to produce more than one phenotype when exposed to different environments

Polymorphisms: Naturally occurring DNA variants among individuals in a population as a result of a spontaneous mutations

Positive selection: The process by which new advantageous genetic variants sweep through a population favoring advantageous alleles toward fixation

Proboscis: The elongated mouthpart of an insect used for food intake

Pseudogenization: An evolutionary process whereby mutations cause a gene to become dysfunctional by disruption of its regulatory or coding sequence

RNAi-mediated inhibition: A process by which expression of a double-stranded RNA activates ribonucleases that degrade homologous mRNA into short fragments.

RNA-seq: Large-scale sequencing of all the RNA, or at least messenger RNA, in a cell, tissue, or animal.

Subfunctionalization: The acquisition of complementary functions of two genes after gene duplication

Sympatric: Occurring in the same geographic area overlapping in distribution

Transcript: The RNA product of a gene

Transposon: A DNA sequence that can move and change its position within a genome

Xenobiotics: Substances that are foreign to the body or to an ecological system

**Figure 1:**
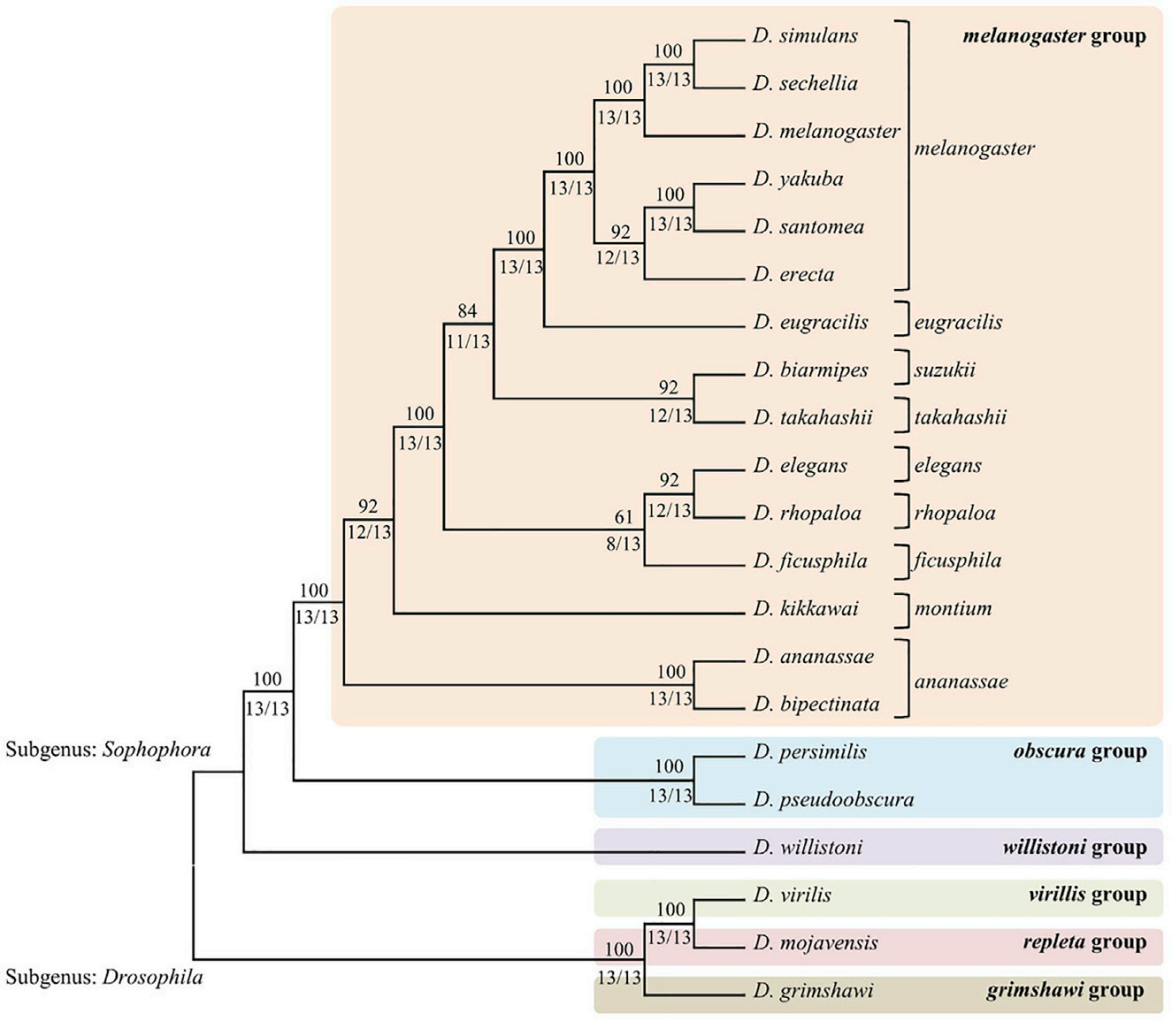
A Phylogenetic Tree Based on Combined Phylogenetic Relationships of 13 Enzymes Numbers above each branch indicate the percentage agreement across all enzymes, and numbers below each branch indicate the number of phylogenetic trees for each individual enzyme that supports the branch. From Seetharam and Stuart, 2013, reproduced under the Creative Commons Attribution License.

The well-studied species *Drosophila melanogaster* offers powerful tools that can be used to genetically dissect complex behaviors. Complete, well-annotated genome sequences for 30 species in the genus *Drosophila* (Drosophila 12 Genomes Consortium, 2007, Song et al., 2011, Miller et al., 2018, Yang et al., 2018, Wiegmann and Richards, 2018) (Figure 1) enable comparative evolutionary studies on complex behaviors. Olfactory behaviors toward food sources, mating partners (Ahmed et al., 2019), *oviposition* site selection (reviewed in Anholt et al., 2020), and avoidance of predators, parasites (Ebrahim et al., 2015), and harmful microbes (Stensmyr et al., 2012) are especially important drivers of behavioral evolution.

In this review I will focus on chemoreceptors as evolutionary substrates for host plant selection. Members of the genus *Drosophila* can be categorized as generalists and specialists. The evolutionary advantage of generalism is the ability to exploit diverse resources for feeding and *oviposition* providing behavioral flexibility under conditions of competition or limited availability of any one resource. Specialists have an advantage in that they can occupy an ecological niche that provides a reliable food source and *oviposition* site but is toxic to related species.

## *Drosophila* Chemosensation

The recognition of chemical signals is essential for the survival and reproductive success of insects, and large and diverse gene families of chemoreceptors have evolved (Box 1). Among insects, *D. melanogaster* has the best-characterized chemoreceptor repertoire (Joseph and Carlson, 2015). The evolutionarily most ancient family of chemoreceptors is represented by the *ionotropic receptors* (IRs) (Benton et al., 2009), which are found in early diverging insects (Archaeognatha and Zygentoma; Missbach et al., 2014) and as far back as Protista (Croset et al., 2010). IRs recognize, among others, water-soluble molecules, including alcohols, amines, and acids (Ai et al., 2010, Min et al., 2013). With the advent of a terrestrial lifestyle, a large and diverse family of gustatory receptors (GRs) that evaluates food and regulates nutrient intake evolved (Scott et al., 2001, Scott, 2018, Robertson, 2019). With the appearance of flying insects, the family of canonical *odorant* receptors (ORs), which recognize airborne odorants, (Robertson et al., 2003, Missbach et al., 2014, Brand et al., 2018) emerged along with the common co-receptor, ORCO. The latter may be ancestral to the ORs and may have been derived from the GR family (Robertson, 2019). ORCO is an obligatory partner for all individual OR receptors and is necessary for their localization to the chemosensory dendritic membrane (Larsson et al., 2004) and for activation by their cognate ligands (Wicher et al., 2008, Sato et al., 2008).

Box 1 **Organization of Olfactory Sensory Neurons in Drosophila melanogaster**

Olfaction in *Drosophila* is mediated through olfactory receptor neurons housed in three types of morphologically distinct sensilla on the third antennal segments and the maxillary palps. Neurons that express members of the *Or* family are housed in basiconic sensilla. There are 10 distinct types of basiconic sensilla on the third antennal segment, designated ab1 through ab10 (de Bruyne et al., 2001, Couto et al., 2005). The ab1 sensilla contains four chemosensory neurons (A–D), and the others each contain two chemosensory neurons, designated A and B, which can be distinguished by their spiking pattern. Neurons housed in the same sensilla types follow a strict pairing rule, so that a neuron with a defined molecular response profile always occurs together with the same partner (de Bruyne et al., 2001). Similarly, each maxillary palp contains 60 basiconic sensilla (pb1–pb3), divided into three sensillar classes, each of which contains an A and a B neuron, again following a strict pairing rule (de Bruyne et al., 1999). The third antennal segment also contains four functional types of coeloconic sensilla, which house neurons that express *Ir* receptors (Yao et al., 2005), and four functional types of trichoid sensilla (at1–at4), which house neurons that respond to pheromones (Miller and Carlson, 2010) (e.g., the *Or67d* receptor expressed in T1 sensilla responds to the pheromone 11-*cis*-vaccenyl acetate; Ha and Smith, 2006). The nomenclature of chemoreceptors designates the family (e.g., *Or* for olfactory receptors); the cytological location on the chromosome, e.g., location 47 is on the right arm of the second chromosome; and the sequence of related family members, e.g., a, b, etc. (Drosophila Odorant Receptor Nomenclature Committee, 2000).

Odorant-binding proteins (OBPs) may have evolved to serve as carriers for hydrophobic ligands in the aqueous perilymph that surrounds olfactory sensory neurons (Pelosi et al., 2014, Larter et al., 2016). However, their precise role in modulating olfactory responses remains unclear. *RNAi-mediated inhibition* of OBPs modulates behavioral responses in a sexually dimorphic manner toward a variety of odorants (Swarup et al., 2011), and deletion of *Obp83a* and *Obp83b* affects deactivation kinetics of olfactory responses (Scheuermann and Smith, 2019). Simultaneous deletion of four highly expressed OBPs retained robust responses against a wide variety of odorants measured electrophysiologically (Xiao et al., 2019). Furthermore, despite their nomenclature, it has become increasingly clear that members of the OBP family also participate in still poorly understood non-chemosensory functions (Findlay et al., 2008, Arya et al., 2010).

Two members of the *D. melanogaster Gr* family, Gr21a and Gr63a, are co-expressed in antennal chemosensory neurons and mediate chemosensation of carbon dioxide (Jones et al., 2007), an *odorant* that can serve both as an alarm signal to trigger avoidance behavior (Suh et al., 2004) and as an attractant because CO_2_ is a by-product of yeast fermentation, a food source for flies. Attractive responses require IR25a, which is a co-receptor for members of the IR family, indicating that different chemosensory modalities may mediate repulsion and attraction to this ecologically relevant *odorant* (van Breugel et al., 2018). Finally, members of the *D. melanogaster* Pickpocket (PPK) ion channel family mediate recognition of water via water-sensing taste sensilla on the *proboscis* that express ppk28 (Chen et al., 2010, Cameron et al., 2010) and courtship pheromones via a channel complex that includes ppk23 expressed on male forelegs (Lu et al., 2012, Toda et al., 2012, Thistle et al., 2012, Mast et al., 2014, Seeholzer et al., 2018).

Although ecologically relevant ligands have not been identified for the majority of chemoreceptors, *molecular response profiles* and ligand specificities of ORs, IRs, and GRs have been characterized using commercially available odorants and tastants (Joseph and Carlson, 2015). Interactions between several OBPs and odorants have also been documented (Kim et al., 1998, Matsuo et al., 2007, Swarup et al., 2011). Sensory neurons that express the same ORs converge on the same output neurons in the antennal lobe of the brain, forming complex synaptic processing units, glomeruli (Vosshall et al., 2000, Grabe et al., 2016). Activation of chemosensory neurons is translated in a pattern of glomerular activity, which is decoded in higher brain regions (Vosshall et al., 2000; Marin et al., 2002; Wong et al., 2002; Masse et al., 2009; Caron et al., 2013; Joseph and Carlson, 2015).

## Rapid Evolution and Diversification of Chemoreceptor Genes

Insect chemoreceptor genes evolve rapidly, leading to extensive diversification and birth and death of gene family members (McBride, 2007, McBride et al., 2007: Sánchez-Gracia et al., 2009, Vieira and Rozas, 2011, Cande et al., 2013). One striking example of ecological adaptations of chemosensory responsiveness comes from a direct comparison between *molecular response profiles* of *D. melanogaster* and *Anopheles gambiae*, which showed that *odorant* recognition has been adapted to the distinct ecological needs of each of these species. Female mosquitoes require a blood meal to produce eggs, whereas *D. melanogaster* feeds on fruit. Odorant perception in *A. gambiae* is tuned predominantly to aromatics found in human sweat, whereas olfactory perceptions in *D. melanogaster* are shaped primarily by esters, which are prevalent in fruit (Carey et al., 2010).

Although the earliest evolutionary origins of the insect chemoreceptor families remain unknown, their evolution likely involved gene duplication and diversification events along with chromosomal rearrangements (Hekmat-Scafe et al., 2002, Nozawa and Nei, 2007). The functions of many members of the *D. melanogaster* chemoreceptor families remain unknown, and the functional consequences of receptor *gene duplication* and subsequent *neo- or subfunctionalization* have remained largely unexplored. *Gene duplication* could relax evolutionary constraint on the daughter genes enabling rapid adaptive evolution. Expansion of chemoreceptor subfamilies can lead to expansion of *molecular response profiles* within the chemosensory recognition repertoire, which might buffer chemosensory ability by generating *functional redundancy* within an expanded family of chemoreceptors. Alternatively, chemoreceptor genes might undergo neofunctionalization and functionally diversify to adopt functions not directly related to the recognition of external odorants or tastants.

One intriguing question is how expression of daughter genes after a duplication event becomes segregated in different olfactory sensory neurons. One can speculate that expression of *Or* genes is silenced and that activation of individual receptors is accomplished through higher-order chromatin conformational modifications, which bring enhancers for specific transcriptional regulators in close proximity to single *Or* genes, as has been shown for regulation of singular olfactory receptor expression in the mouse (Monahan et al., 2017 and 2019). In *Drosophila*, combinatorial usage of transcription factors during development appears to play a prominent role in receptor choice determination (Jafari et al., 2012, Barish and Volkan, 2015). However, the mechanisms that regulate expression of singular *Or* genes in insects remain to be further clarified.

One example of evolutionary diversification is evident in the family of IRs, where IR40a and IR93A along with the common IR25a co-receptor have become specialized for humidity sensation (Enjin et al., 2016, Knecht et al., 2017). In addition to members of the IR family, an OBP, OBP59a, has also been implicated in humidity sensing (Sun et al., 2018). IR93a and IR25a, along with IR21a, also mediate temperature sensation and are expressed in thermosensory neurons in the *arista* (Enjin et al., 2016, Budelli et al., 2019). In addition, a member of the GR family, GR28b, has been identified as a peripheral thermosensor that responds to rapid warming (Ni et al., 2013). It is of interest that IR25a has been implicated in temperature-dependent regulation of the *circadian* clock (Chen et al., 2015).

Another example of evolutionary diversification of chemoreceptors comes from the large IR20a clade of IR genes. Members of this clade are expressed in diverse gustatory neurons in the labellum, the pharynx, the wing margin, and the front legs. This clade includes 35 genes with on average 16% sequence identity and 7 genes with premature stop codons, which bears testimony to their rapid evolution (Koh et al., 2014). Comparisons of sequence variation of members of this clade among the sequenced inbred wild-derived lines of the *Drosophila melanogaster* Genetic Reference Panel (Huang et al., 2014, Mackay et al., 2012) with divergence between *D. melanogaster* and *Drosophila simulans* showed evidence for positive selection among the related *paralogs Ir52c* and *Ir52d* (Koh et al., 2014). These genes are expressed in neurons on the forelegs of *D. melanogaster* males and are associated with mating behavior, possibly through recognition of pheromones (Koh et al., 2014).

Other examples of likely sub- and neofunctionalization are evident among paralogs of a cluster of *Obp* genes on the *D. melanogaste*r X chromosome. Association analyses in wild-derived inbred *D. melanogaster* lines showed polymorphisms in *Obp19a* and *Obp19b* associated with variation in behavioral responses to benzaldehyde, whereas *Obp19c* harbored a SNP associated with variation in behavioral response to hexanal (Arya et al., 2010). In addition, two polymorphic markers in *Obp19d* were associated with variation in lifespan. In the antenna, *Obp19a* is expressed in a subset of basiconic sensilla, whereas *Obp19d* is associated with extrasensillar uninnervated spinules (Larter et al., 2016). *Gene ontology enrichment analyses* of ensembles of coregulated genes with each of the focal genes implicated *Obp19c* in *oviposition* and postmating behavior (Arya et al., 2010). In this light, it is of interest that *Obp19c* is also expressed in ovaries. Furthermore, *Obp8a* on the *D. melanogaster* X chromosome shows high expression in the male *accessory gland*, suggesting that *Obp8a* and *Obp19c*, and potentially other OBPs found in seminal fluid (Findlay et al., 2008), may bind thus far unidentified hydrophobic molecules associated with the transfer of sperm during mating and stimulation of *oviposition*.

## Co-evolution of Chemosensation and Host Plant Selection

Adaptation to host plants on which flies oviposit and on which larvae can develop depends to a large extent on chemosensation. Such adaptation has been extensively studied in *Drosophila sechellia*, which prefers to feed on *Morinda citrifolia* fruit, which is avoided by its sister species *D. simulans* (Jones, 2005). The ability to feed on a food source that is toxic to competing species ensures survival. *M. citrifolia* produces hexanoic and octanoic acids, fatty acids to which *D. sechellia* are attracted and that are toxic and repellent to other *Drosophila* species (Amlou et al., 1998). This specialization is accompanied by rapid evolutionary changes in the chemoreceptor repertoire of *D. sechellia* with accumulation of *loss-of-function alleles*, especially among the *Gr* family (McBride, 2007, McBride et al., 2007). One well-characterized change in the chemoreceptor repertoire involves *Obp57d* and *Obp57e,* which are expressed in cells in the tarsi (Matsuo et al., 2007). Expression of these OBPs is controlled by conserved *cis regulatory elements* (Tomioka et al., 2012). A 4-bp CCAT insertion upstream of the *D. sechellia Obp57e* gene prevents its expression, even though its open reading frame is intact (Matsuo et al., 2007). In addition, a premature stop codon in the *D. sechellia Obp56e* gene has generated a loss-of-function allele (Dworkin and Jones, 2009). Thus, multiple *Obp* alleles have evolved in *D. sechellia* that together culminate in facilitating host preference behavior by preventing taste avoidance of hexanoic and octanoic acid. When *Obp57e* and *Obp57d* in *D. melanogaster* were deleted, behavioral responses to hexanoic and octanoic acids also changed (Matsuo et al., 2007). Hybrids between *D. melanogaster Obp57d/e knockout* flies and its closely related sister species *D. simulans* or *D. sechellia* shifted oviposition site preferences to that of either the *D. simulans* or *D. sechelli*a parent (Matsuo et al., 2007). In addition, gene expression studies identified several other genes that showed extensive upregulation in *D. sechellia* compared with its sister species *D. simulans*, including *Or22a* (Kopp et al., 2008), *Obp50a*, *Or85c,* and *Ir84a* (Shiao et al., 2015). Interestingly, on the island of Mayotte off the East Coast of Africa, an isolated population of *Drosophila yakuba*, commonly considered a generalist species, independently evolved specialization for *M. citrifolia*, similar to *D. sechellia*, providing an example of recurrent evolutionary adaptation (Yassin et al., 2016).

Although cosmopolitan *D. melanogaster* are considered generalist feeders, they prefer to lay eggs on citrus substrates, which produce terpenes that are detected via the OR19a receptor (Dweck et al., 2013). Studies on wild populations of *D. melanogaster* in Zimbabwe show that these African flies feed and oviposit almost exclusively on marula fruit (*Sclerocarya birrea*), a citrus-like endemic fruit. This specialization is not observed in *sympatric D. simulans* (Mansourian et al., 2018). Ethyl isovalerate produced by the marula fruit acts as an olfactory cue for oviposition site preference by activating ab3A neurons, which project to the DM2 glomerulus and express a distinct *Or22a/Or22b* variant in this fly population. Even laboratory-reared flies of the *Canton-S* strain still favor marula fruit in preference assays (Mansourian et al., 2018). Thus, specialization on marula of African *D. melanogaster* may be ancestral to the generalist host plant relationships of cosmopolitan *D. melanogaster*, illustrating *plasticity and evolvability* of insect-host plant relationships.

A similar example of host specialization comes from *Drosophila erecta*, another close relative of *D. melanogaster*, endemic in forests of west central Africa. *D. erecta* has evolved a specialized relationship with screw pine fruits (*Pandanus sp.*). These fruits produce 3-methyl-2-butenyl acetate. The proportion of olfactory sensory neurons that respond to this odorant (ab3A neurons) has increased by ∼40% in *D. erecta* with a concomitant ∼2.5-fold increase in volumes of its corresponding glomeruli in the antennal lobes. Exposure to 3-methyl-2-butenyl acetate induces egg laying in *D. erecta*, but not in *D. melanogaster* (Linz et al., 2013).

One of the best studied examples of host plant adaptations in the genus *Drosophila* comes from *Drosophila mojavensis*, which feeds on decomposing cactus in Arizona, the Mojave desert and Baja California, the Sonoran Desert, and Catalina Island. Different races of *D. mojavensis* have developed specialized host plant relationships with different cacti that are endemic at each location (Newby and Etges, 1998). Olfactory adaptations to distinct odorants emanating from each cactus species have been characterized both electrophysiologically and through behavioral studies (Date et al., 2013). In addition, analyses of genome-wide transcript abundances showed differential expression of members of the *Or* gene family between the different *D. mojavensis* populations (Crowley-Gall et al., 2016). Flies from the Mojave desert showed significant upregulation of several OR genes compared with flies from Catalina Island, notably Or67b and Or71a, which are expressed in ab9B and pb1B neurons, respectively, and are excited by aromatics. Differential expression of receptors was correlated with differential activity, measured electrophysiologically, and reflected in differences in the proportions of specific olfactory sensory neurons, in which they are expressed (Crowley-Gall et al., 2016).

Different habitats and different host plant specializations among the *D. mojavensis* populations could ultimately lead to reproductive barriers (Pfeiler et al., 2009). Analyses of genome sequences between *D. mojavensis* and its close relatives *D. arizonae* and *D. navojoa* revealed chromosomal inversion differences that form a barrier to interbreeding (Sanchez-Flores et al., 2016), although host plant specialization may not necessarily have been the driving force for this reproductive isolation.

Olfactory adaptations can have economic consequences. *Drosophila suzukii* has emerged as a major agricultural pest during the last decade as it has spread from Southeast Asia to Europe and North America (Walsh et al., 2011). Most *Drosophila* species are attracted to decaying fruit, whereas *D. suzukii* females oviposit on ripening fruit. *D. suzukii* females have evolved an enlarged serrated ovipositor, which enables them to penetrate the soft skin of ripe fruit, for example, a variety of berries (Atallah et al., 2014). Behavioral studies show that *D. suzukii* is attracted to the odor of ripe strawberry and that oviposition behavior on ripe fruit is reduced when the common odorant co-receptor gene, *Orco*, is knocked down by RNAi or eliminated through CRISPR deletion (Karageorgi et al., 2017).

In contrast to its closely related species *Drosophila biarmipes,* the *Or* gene repertoire of *D. suzukii* has undergone duplications at the *Or23a* and *Or67a* loci and there is evidence for positive selection at the *Or67a* locus (Hickner et al., 2016). Elegant experiments in *D. melanogaster* in which different ORs were ectopically expressed in sensilla that lack expression of the endogenous Or22a receptor showed that the Or67a receptor could be activated strongly by methyl benzoate and ethyl benzoate, both of which have fruity odor qualities (Hallem and Carlson, 2006). However, volatiles arising from berries are complex; e.g., strawberries exude as many as 147 volatiles (Kim et al., 2013), making assessment of causal attractants challenging. *D. suzukii* also responds to the leaf odor β-cyclocitral, which does not elicit responses from its close relatives *D. biarmipes* and *Drosophila takahashii*. The responses to β-cyclocitral are mediated via the A neurons of ab3 sensilla (Keesey et al., 2015). Thus, both fruit volatiles and leaf volatiles may play a role in feeding and oviposition site selection by *D. suzukii*. Further examination of the *Or* gene repertoire in *D. suzukii* revealed *pseudogenization* of *Or74a*, *Or85a,* and *Or98b*. B neurons of the ab2 sensilla, which express *Or85a,* in *D. melanogaster* and *D. biarmipes* respond strongly to the fruity odorant ethyl 3-hydroxybutyrate, these neurons in *D. suzukii* are not responsive to this odorant (Keesey et al., 2015). Thus the functional significance of pseudogenization of this gene and other genes in *D. suzukii* remains to be established (Hickner et al., 2016).

**Figure 2:**
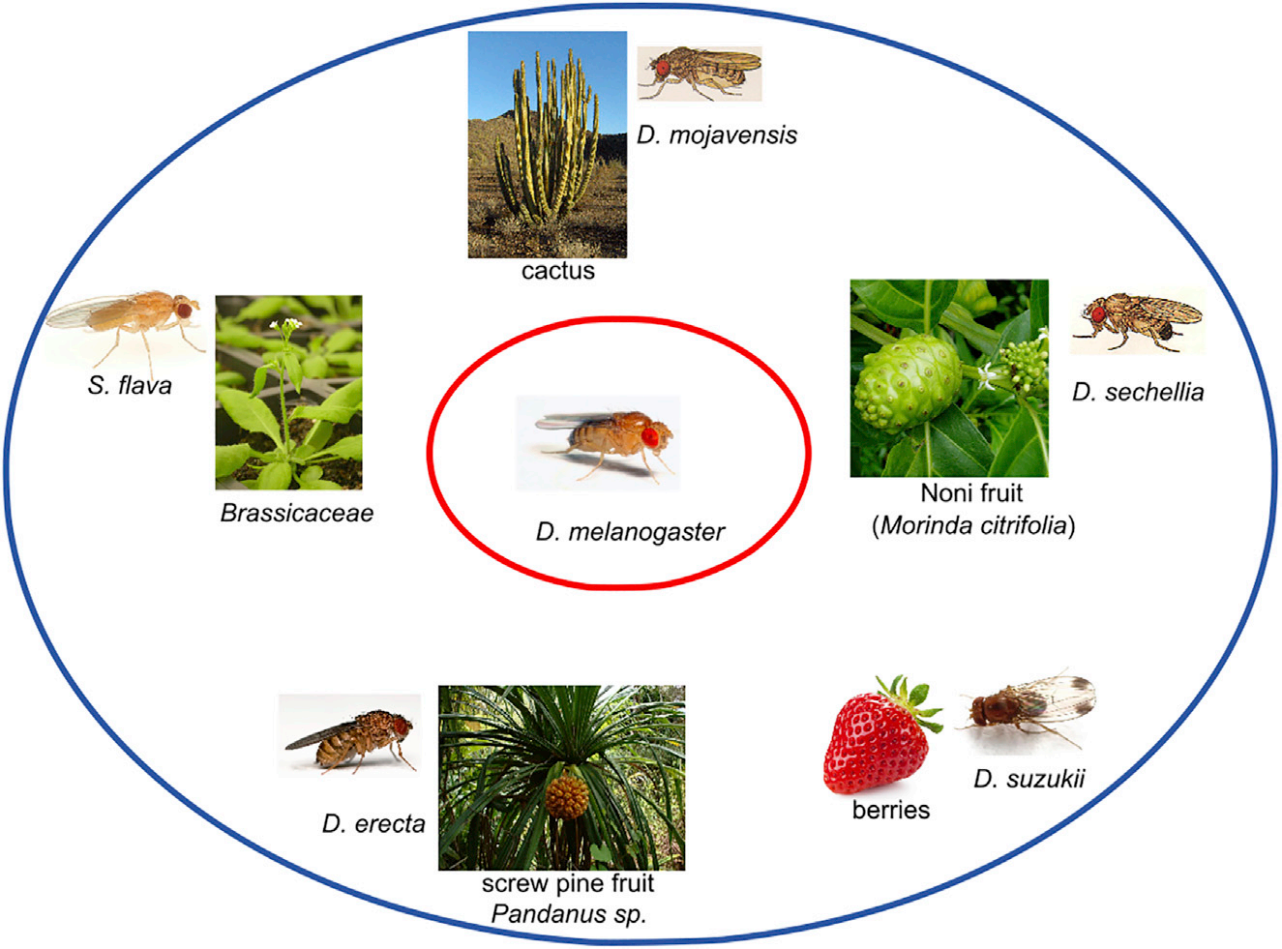
Illustration of the Diversity of Host Plant Specializations among Drosophilids Different specializations have developed independently along different branches of the *Drosophila* phylogeny. The most extensively studied member of the genus, *D. melanogaster,* is depicted in the center. Although cosmopolitan *D. melanogaster* are considered generalist, this generalism may have evolved from specialization on marula fruit of ancestral African populations (Mansourian et al., 2018).

Another adaptation driven by chemosensation is exemplified by the leaf-mining drosophilid *Scaptomyza flava*, which oviposits, and its larvae feed on leaves of the family Brassicaceae, which includes *Arabidopsis thaliana* (Whiteman et al., 2011, Goldman-Huertas et al., 2015). Evolution of *S. flava* herbivory has been accompanied by extensive changes in its OR repertoire with pseudogenization of multiple *Or* genes that respond to short-chain aliphatic esters, commonly found in yeast. The most striking change in the *Or* repertoire in this species is duplication at the *Or67b* locus, which has given rise to three paralogs with evidence for *positive selection* (Goldman-Huertas et al., 2015). It is possible that positive selection at this locus is intimately associated with feeding behavior on plant leaves, because its counterpart, the *D. melanogaster OR67b* receptor, responds to the green-leaf volatile (Z)-3-hexenol (Galizia et al., 2010). The diversity of specializations within the genus *Drosophila* is illustrated in Figure 2. Finally, it should be noted that the absence of toxicity and lack of preference for a potential food source in the wild does not necessarily imply inability *per se* to feed and develop on that food source. For example, in the absence of screw pine fruits, *D. erecta* can feed on fungi, *Ficus capensis* fruits (Lachaise and Tsacas, 1974), and even on bananas (Rio et al., 1983).

## Co-evolution of Neural Projections with Development of Preference for Chemosensory Cues

Development of preference for chemical cues from host plants is accompanied by alterations in the functional organization of the fly's olfactory system. The *D. sechellia* antennae respond to femtogram quantities of methyl hexanoate, produced by its host plant, and this extraordinary sensitivity is reflected by an approximately 3-fold overrepresentation of neurons responding to this odorant compared with *D. melanogaster* (Dekker et al., 2006). Overrepresentation of this neuronal population is accompanied by a corresponding increase in volume of the glomerulus to which they project (Dekker et al., 2006). The A neuron of the ab3 sensillum responds to hexanoate esters and projects to its corresponding enlarged DM2 glomerulus, whereas B neurons in the ab3 sensilla respond to 2-heptanone, which is also produced by the Morinda fruit, and the glomerulus to which these neurons project is also enlarged (Ibba et al., 2010). Furthermore, a single amino acid change in the *D. sechellia* IR75b receptor, which is expressed in *the* ac3 sensilla, confers sensitivity and attraction to hexanoic acid (Prieto-Godino et al., 2017). This amino acid substitution and the resulting change in odorant response profile is accompanied by expansion of the DL2d glomerulus, which receives projections from IR75b-expressing neurons. However, no neuroanatomical changes were observed in higher-order circuits (Prieto-Godino et al., 2017). Thus, adaptations to specific olfactory cues that mediate host plant specialization can be accompanied by overrepresentation of olfactory receptor neuron populations and their projections to the antennal lobes.

## Co-evolution of Cytochrome P450s during Host Plant Adaptation

**Figure 3:**
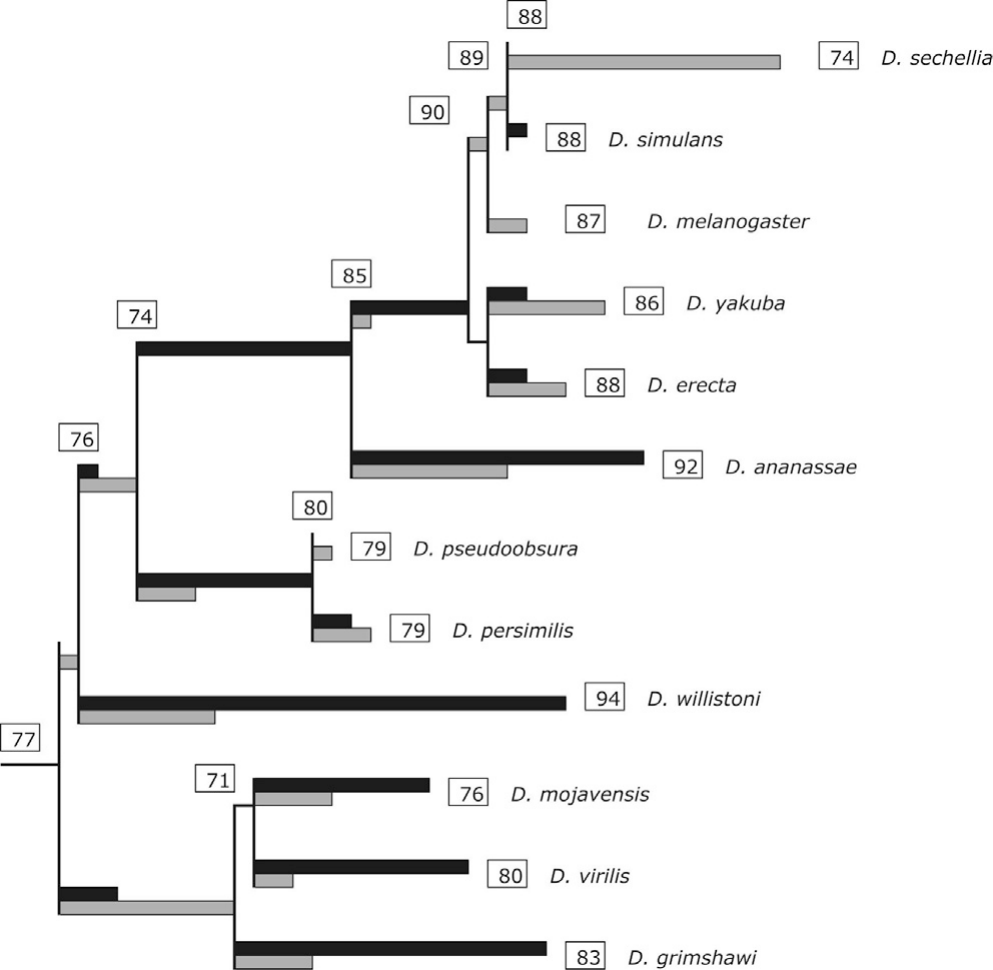
Evolution of the Family of Cytochrome P450 Genes among Drosophilids, Illustrating Gains and Losses on the Topology of the Phylogeny of 12 *Drosophila* Species The lengths of the black and gray bars are proportional to the number of gene gains and losses in each lineage, respectively. The number of functional cytochrome P450 genes in each species is indicated in the boxes along with the number of inferred ancestral cytochrome P450 genes. From Good et al., 2014, reproduced under the Creative Commons CC-BY-NC license.

Specialized adaptations of insects to host plants depend not only on chemosensation but also require mechanisms that can neutralize toxic substances that plants produce to defend against herbivory. Members of the cytochrome P450 family play a major role in detoxification of *xenobiotics*. Cytochrome P450s are a diverse class of enzymes that perform a variety of functions from synthesis and degradation of ecdysteroids and juvenile hormone to the processing of various toxic chemicals insects may encounter in their environments (Feyereisen, 1999). Rapid evolution of the large family of cytochrome P450s, driven by gene duplications and diversification, accompanies olfactory adaptations to host plant specializations (Wu et al., 2011, McDonnell et al., 2012, Good et al., 2014, Harrop et al., 2014). Analysis of the cytochrome P450 gene family along the evolutionary trajectory of 12 *Drosophila* species has detected 114 gene gains and 74 gene losses (Figure 3) (Good et al., 2014). The cytochrome P450 gene family in *D. melanogaster* encompasses 90 genes, of which 83 encode functional transcripts, most of which belong to the CYP4 and CYP6 families (Tijet et al., 2001). Duplication of the *Cyp6g1* gene has occurred at least four times in the *Drosophila* lineage, and *Cyp6g1* paralogs are associated with insecticide resistance both in *D. melanogaster* and its sister species *D. simulans* (Harrop et al., 2014). Furthermore, *copy number variants and transposon* insertions at the 5′ regulatory region of the *D. melanogaster Cyp6g1* locus have been associated with increased transcription of *Cyp6g1* (Daborn et al., 2002) and resistance of *Cyp6g1* alleles to dichlorodiphenyltrichloroethane in field populations (Schmidt et al., 2010). Strong directional selection in a California population of *D. simulans* has resulted in fixation of a Doc transposable element in the 5′ flanking region of Cyp6g1, which is also associated with increased transcription (Schlenke and Begun, 2004).

Molecular modeling of the effects of allelic variants of cytochrome P450s on protein folding, based on structural information from mammalian cytochrome P450s, shows that changes in protein conformation can have diverse effects on catalytic activity across different insect species. Phylogenetically closely related CYP450s can display distinct substrate specificities, whereas distantly related CYP450s may act on similar substrates. These studies also provided insight into the range of specificities of different members of the CYP450 family, ranging from narrow to broad (Schuler and Berenbaum, 2013).

Alkaloid-metabolizing P450 enzymes have been implicated in host adaptations of the cosmopolitan species *D. hydei* (Danielson et al., 1997) and in cactophilic *Drosophila* (Danielson et al., 1995). Two cytochrome P450 genes, *Cyp28a1* and *Cyp4d10*, have evolved to detoxify alkaloids from cactus host plants to enable host plant utilization by the cactophilic species *Drosophila mettleri* (Bono et al., 2008). Thus, evolutionary specialization on host plants may have been facilitated by adaptive tuning of the chemosensory gene repertoire along with evolution of a spectrum of cytochrome P450s targeted toward detoxification of potentially harmful or aversive plant-derived xenobiotics.

The adaptive mechanisms that enable co-evolution of different members of the chemoreceptor repertoire (e.g., OBPs and ORs) and detoxification enzymes remain poorly understood. One can hypothesize that selection of alleles of members of the cytochrome P450 family might occur first to enable a generalist species to access a previously unavailable food source, which would result in a selective advantage, followed by adaptation of chemoreceptors. However, in the absence of clear evidence this hypothesis remains speculative.

## Concluding Remarks

Evolution of specialization requires co-evolution of multigene families of chemoreceptors and detoxification enzymes concomitant with modifications of neural circuitry in the brain. This complex process raises questions about the evolutionary mechanisms and adaptive forces that drive the acquisition of behavioral specializations (Box 2). The vast amount of information and resources available for Drosophilids make this genus an excellent model system to explore the evolutionary ecology of behavioral diversification.

Box 2 **Outstanding Questions**

A central question related to evolution of the chemoreceptor repertoire is how evolution of the odorant receptor repertoire is accommodated in the neural projection to the antennal lobe. Does the total number of olfactory sensory neurons increase or does expansion of one neuronal specificity and enlargement of a single glomerulus occur at the expense of others? Do the same OR-expressing sensory neurons target the same glomeruli in all Drosophilids? Is it possible that corresponding olfactory sensory neurons in some species express different ORs tuned to their relevant host plant?

Finally, occupation of a specialized niche for oviposition and feeding establishes a reproductive barrier. Thus, host plant adaptation is intimately associated with speciation. How host plant adaptation functions in the transition from prezygotic to postzygotic isolation remains an area of current interest.

The genus *Drosophila* provides an ideal system to address these questions.
